# A Century of Shope Papillomavirus in Museum Rabbit Specimens

**DOI:** 10.1371/journal.pone.0132172

**Published:** 2015-07-06

**Authors:** Clara Escudero Duch, Richard A. J. Williams, Robert M. Timm, Javier Perez-Tris, Laura Benitez

**Affiliations:** 1 Department of Microbiology III, Faculty of Biological Sciences, Universidad Complutense de Madrid, Madrid, Spain; 2 Department of Zoology and Physical Anthropology, Faculty of Biological Sciences, Universidad Complutense de Madrid, Madrid, Spain; 3 Natural Sciences, Saint Louis University, Madrid, Spain; 4 Department of Ecology and Evolutionary Biology & Natural History Museum, University of Kansas, Lawrence, Kansas 66045, United States of America; Natural History Museum of Denmark, DENMARK

## Abstract

*Sylvilagus floridanus* Papillomavirus (SfPV) causes growth of large horn-like tumors on rabbits. SfPV was described in cottontail rabbits (probably *Sylvilagus floridanus*) from Kansas and Iowa by Richard Shope in 1933, and detected in *S*. *audubonii* in 2011. It is known almost exclusively from the US Midwest. We explored the University of Kansas Natural History Museum for historical museum specimens infected with SfPV, using molecular techniques, to assess if additional wild species host SfPV, and whether SfPV occurs throughout the host range, or just in the Midwest. Secondary aims were to detect distinct strains, and evidence for strain spatio-temporal specificity. We found 20 of 1395 rabbits in the KU collection SfPV symptomatic. Three of 17 lagomorph species (*S*. *nuttallii*, and the two known hosts) were symptomatic, while *Brachylagus*, *Lepus* and eight additional *Sylvilagus* species were not. 13 symptomatic individuals were positive by molecular testing, including the first *S*. *nuttallii* detection. Prevalence of symptomatic individuals was significantly higher in *Sylvilagus* (1.8%) than *Lepus*. Half of these specimens came from Kansas, though new molecular detections were obtained from Jalisco—Mexico’s first—and Nebraska, Nevada, New Mexico, and Texas, USA. We document the oldest lab-confirmed case (Kansas, 1915), pre-dating Shope’s first case. SfPV amplification was possible from 63.2% of symptomatic museum specimens. Using multiple methodologies, rolling circle amplification and, multiple isothermal displacement amplification in addition to PCR, greatly improved detection rates. Short sequences were obtained from six individuals for two genes. L1 gene sequences were identical to all previously detected sequences; E7 gene sequences, were more variable, yielding five distinct SfPV1 strains that differing by less than 2% from strains circulating in the Midwest and Mexico, between 1915 and 2005. Our results do not clarify whether strains are host species specific, though they are consistent with SfPV specificity to genus *Sylvilagus*.

## Introduction

Rabbit papillomatosis is a viral infection caused by *Sylvilagus floridanus* papillomavirus (SfPV; family *Papillomaviridae*). It can cause extensive skin growths in cottontail rabbits, especially around the head. Although long known to hunters, the disease was first reported in 1931 [1], and described by Richard E. Shope in 1933 as the first papillomavirus (PV) [2]. It is now known as Shope papillomavirus, cottontail rabbit papillomavirus (CRPV) [3], and Kappapapillomavirus 2 [4]; the last has been accepted by the International Committee for Virus Taxonomy [5]. SfPV growths frequently occur on the face, head, neck, and back of *Sylvilagus* rabbits, but may appear anywhere (on the affected animal) and can be quite cryptic when small. Growths can be several centimeters in length. SfPV was the first virus confirmed to cause cancer in mammals [6], and has been used in cancer research since the 1930s [7,8]. It remains one the best known in vivo models to study viral infection and the course of papillomavirus diseases from infection to malignancy [7].

Natural infections occur in the North American eastern cottontail rabbit, *Sylvilagus floridanus* [9]. However, most lab confirmed cases [2,10,11] are actually described as *Sylvilagus* sp. or spp. These are almost certainly derived from *S*. *floridanus* based on known distributions. SfPV has recently been described in the desert cottontail, *S*. *audubonii* [12]. The host range may be broader than these two species. Experimental infection can induce cutaneous papillomas in domestic rabbits (*Oryctolagus cuniculus domesticus*), black-tailed jackrabbits (*Lepus californicus*), and snowshoe hares (*Lepus americanus*) [9].

**Fig 1:**
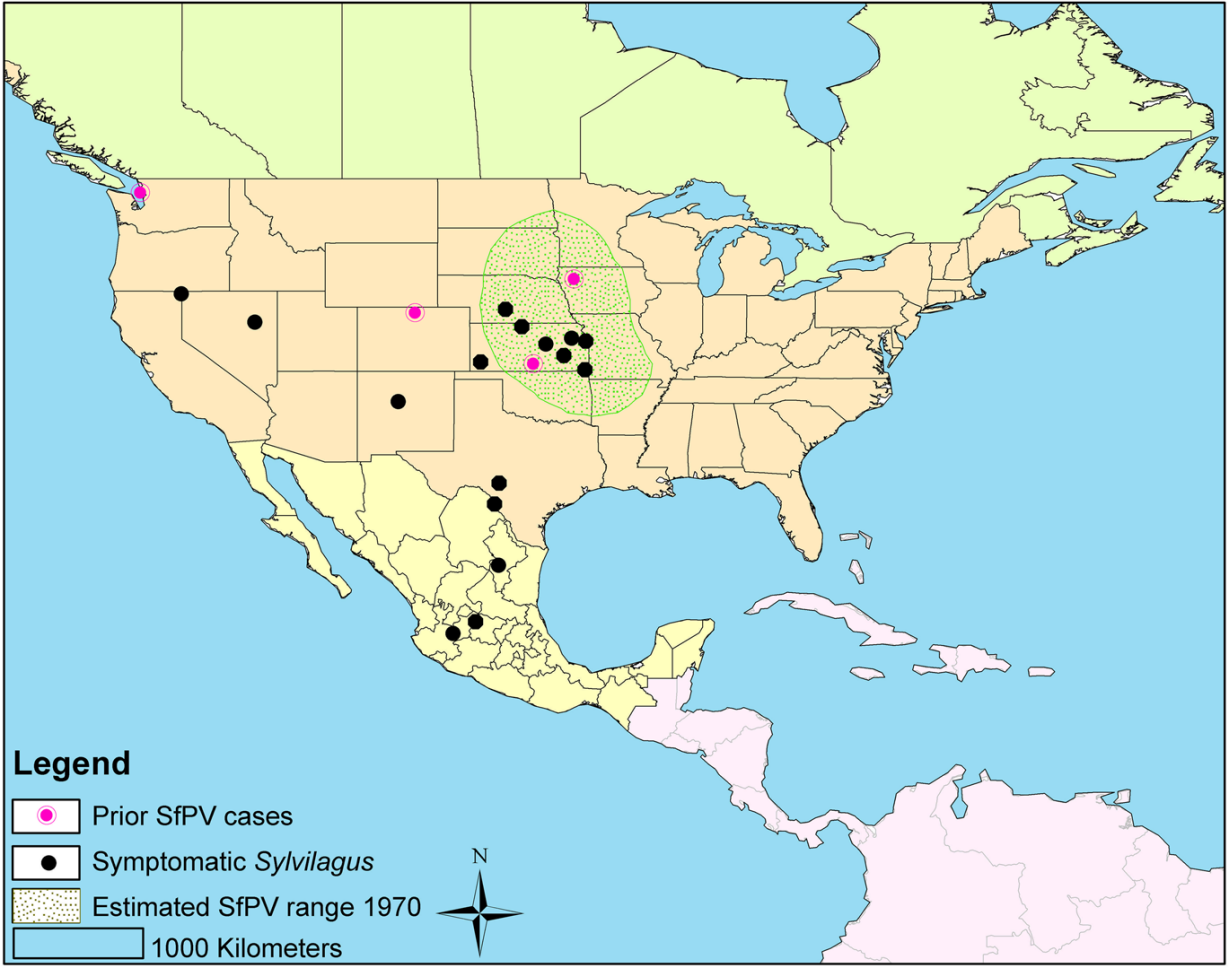
Distribution of naturally occurring Shope papillomas of cottontail rabbits as described by Shope. Redrawn following Kreider, 1981 [13]. The locality of prior PCR positive samples (pink dots) and 16 of 18 localities of symptomatic samples tested in this study (black dots) for which geographical information was available are shown. Three samples were collected from the same locality (Lexington, Nebraska).

SfPV is an enzootic disease of the genus *Sylvilagus* estimated, by Shope in 1980 [13], to occur only in the Midwestern USA (**Fig 1**). It has been lab confirmed from Colorado [10,12]—beyond Shope’s estimated range—Iowa and Kansas [2,11], and Washington State [14]. Symptomatic *S*. *floridanus* have been observed in Minnesota, Missouri, Nebraska, and Oklahoma (RM Timm, unpubl. data). *S*. *floridanus* and, to a lesser extent, *S*. *audubonii*, from west of the Missouri River, especially Kansas, were widely shipped around the U.S. to provide hunters with additional game species, providing a potential route for SfPV dispersion. It seems likely that millions of rabbits were shipped around the USA in this way; Maryland alone, released 207,000 authorized cottontails between 1922 and 1950 [15]. The only documented occurrence of SfPV west of the Rocky Mountains are in a population of *S*. *floridanus* imported to Whidbey Island, Washington, predominantly from Kansas [14]; eastern cottontails did not occur on this island previously, so the SfPV strain found there was almost certainly introduced to the Island along with the eastern cottontails.

The genus *Sylvilagus* is found throughout North and Central America and much of the northern half of South America. Seven species of *Sylvilagus*, including the two known hosts, occur in the Midwestern and western USA [16,17]. *S*. *floridanus* is widespread in southern Canada, the eastern and Midwestern USA, and throughout Central America to northern Costa Rica with disjunct populations in northern South America. *S*. *audubonii* occurs in the southwestern quarter of the continental U.S. and much of Mexico. There are areas in which both species overlap. The known range of SfPV thus constitutes a small portion of its known host range. It remains unclear whether SfPV should be anticipated, at least, throughout the range of known hosts, or whether unidentified ecological factors (e.g., vector distribution, rainfall, etc.) limit SfPV distribution.

All known SfPV sequences share a high degree of identity and are genotype SfPV1 [18]. We use SfPV generically, and SfPV1 specifically where the genotype is known. To date, four complete genomes have been sequenced for SfPV1. Three are 99% identical; CRPV strains Shope [19], Hershey [20], and a4 (CRPVa4) [21]; hereafter collectively referred to as SfPV1a. One additional SfPV1 subtype genome [21] (CRPVb; hereafter SfPV1b), has 97% sequence identity to the original type (strain Shope). The relation between strain and host species is ambiguous. One of three SfPV1a detections is unambiguously from *S*. *floridanus* [20]. The other two hosts [19,21] are likely *S*. *floridanus* if locality data are accurate. One of two SfPV1b detections is unambiguously from *S. audubonii [12].* The other is likely *S*. *floridanus* if locality data are accurate [11].

Traditionally, PVs have been considered as viruses that have co-evolved with their host species, with well-defined papilloma taxa (“genera”) clustered within recognized vertebrate family or higher taxa. For instance, most alphapapillomaviruses are found in *Homo sapiens*, the exceptions being other primate hosts. Similarly, all lambdapapillomaviruses have been detected in Carnivora, all deltapapilloviruses in Artiodactyla, etc. [4]. A number of authors have suggested that papillomaviruses are host species specific, or restricted to closely related species within the same genus [22–26]. As such, their nomenclature is based on the first host from which the virus was isolated [4], e.g. SfPV1—*Sylvilagus floridanus* Papillomavirus 1. PV co-evolution with hosts remains contentious [27], challenged by data from experimental infection, evidence of heterologous PV infection, and the existence of polyphyletic lineages for PVs in several taxonomic groups [27]. PVs have been shown to infect closely related species in lab infection studies (e.g., SfPV in Leporidae [9]), and also in the wild, e.g. the bats *Eptesicus serotinus* and *E*. *isabellinus* bat PV [27]. One bovine PV genotype, BPV-2, has been detected in a number of distantly related hosts. [28,29]. However, the two PV genotypes known from rabbits (SfPV1 and OcPV1, *Oryctolagus cuniculus* papillomavirus, previously known as Rabbit Oral Papillomavirus, ROPV) [4,30] are closely related and are the only recognized members of the genus: *Kappapapillomavirus* 2 and *Kappapapillomavirus* 1, respectively.

Current research efforts primarily focus on detecting novel PV genotypes as our knowledge of the diversity of non-human PVs is incomplete [27]. At present over 280 different PV types have been fully sequenced and placed in 35 genera. Most known PV types are from humans; approximately a third infect non-human species [31], although the true diversity of non-human PV types likely outnumbers that of human PV types [32]. Numerous complete or partial sequences of isolates of bovine, canine, or primate PV have been deposited in databanks (primarily GenBank). The use of tissue archive and museum samples contributes significantly to improve the identification and characterization of diverse pathogens, including viruses, in wild populations (see review [33]). There are few examples of PV detection in archived DNA; human PV has been amplified from a 16th century mummy [34] and avian PV has been detected in a biopsy from a museum specimen collected pre-1936 [35].

**Fig 2:**
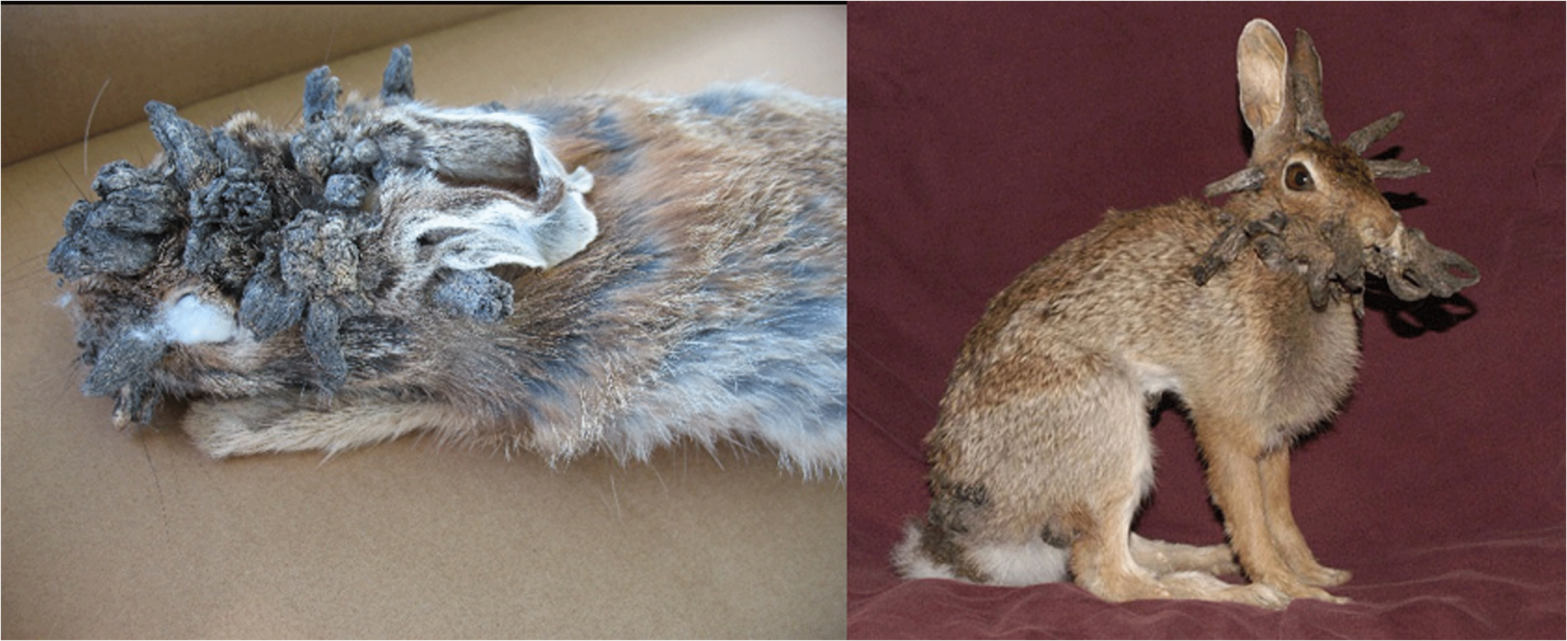
*Sylvilagus floridanus* voucher specimens with typical SfPV horn-like growths. Left, one of the 13 PCR positive specimens; right, specimen not tested to preserve its integrity. Specimens from the University of Kansas Natural History Museum (KU) collection.

The University of Kansas Natural History Museum (KU), Lawrence, Kansas, USA, contains 1395 voucher specimens of rabbits, jackrabbits, and hares (family Leporidae; 17 species, 3 genera) including several specimens of *Sylvilagus* with growths symptomatic for SfPV (**Fig 2**). The aim of this study is to document presence of SfPV in symptomatic KU specimens using molecular techniques, to assess if SfPV is found only in known hosts, or if it has additional natural hosts, and whether SfPV occurs throughout the host range, or just in the Midwest. We highlight methods to increase rates of PV DNA recovery from museum voucher specimens, which may be complicated if viral DNA has degraded due to age or possible chemical exposure. Genetic sequences may reveal whether virus populations have changed during the course of the last century. Museum vouchers can answer a number of questions about SfPV evolution, such as whether few or many SfPV strains exist, whether there is temporal variation in circulating virus strains, if the relative prevalences vary at different times in the past, and whether strains show host specificity.

## Materials and Methods

### Ethics statement

Specimens studied during this research form part of the Mammal Collection of the Natural History Museum, University of Kansas, Lawrence (KU), and were inspected on site by KU Mammal curator, RMT, and RAJW. Additional specimens in the Smithsonian Institution, Washington, D.C. were examined by RMT.

### Samples

All voucher specimens of family Leporidae (*Brachylagus N* = 13, 1 sp.; *Sylvilagus N* = 1096, 11 spp.; *Lepus N* = 286, 5 spp.) deposited at KU, were inspected for the presence of cutaneous lesions consistent with papillomatosis; all specimens were inspected visually and palpated. Samples were collected from all symptomatic specimens detected, except one model specimen regularly used for exhibition. A portion of skin was also collected from one asymptomatic *S*. *floridanus* to serve as negative control. KU catalog numbers for all specimens tested in this study are provided in **Table 1**. The KU collection of leporids dates from the 1890s to the present day, and is particularly well-represented by specimens from Kansas, the Midwestern U.S., and Mexico. The samples, all skin tumors, were excised from the animal and stored dry at 4°C. Sterile scalpel blades and fresh gloves were used to for the collection of each sample.

**Table 1:** Results of tests on *symptomatic* Sylvilagus rabbits. Includes locality and year of host collection.

Species	KU NHM catalog #	Number	Locality	Year	conc. of total nucleic acids (ng/μl/mg)	RCA	PCR L1	PCR E7	MDA+PCR L1
*S*. *floridanus*	2551	7R	Cherokee County, KS, USA	1915	24.61	(+)	(+)	(+)	NT
*S*. *nuttallii*	45843	19R	Willow Creek, NV, USA	1929	90.69	(−)	(−)	(−)	(−)
*S*. *floridanus*	52814	10R	Hamilton, KS, USA	1945	57.64	NT	(−)	(−)	(+)
*S*. *audubonii*	23615	17R	No data	1948	102.85	NT	(−)	(−)	(−)
*S*. *nuttallii*	52813	20R	Bidwell, NV, USA	1949	11.46	NT	(−)	(−)	(+)
*S*. *floridanus*	147300	5R	Rock Springs, TX, USA	1950	2.53	NT	(−)	(−)	(+)
*S*. *floridanus*	52236	8R	Eagle Pass, TX, USA	1953	32.91	NT	(−)	(−)	(+)
*S*. *floridanus*	62403	12R	East Zapotlanejo, Jal, Mexico	1954	170.38	NT	(−)	(−)	(−)
*S*. *floridanus*	63766	1R	Smith Center, KS, USA	1955	140.54	NT	(+)	(+)	NT
*S*. *floridanus*	63762	9R	Carlton, KS, USA	1955	78.36	NT	(−)	(−)	(−)
*S*. *floridanus*	in process	13R	Olathe, KS, USA	1957	22.15	(−)	(+)	(+)	(+)
*S*. *floridanus*	98894	14R	Sierra Potosi, N.L., Mexico	1964	116.64	NT	(−)	(−)	(−)
*S*. *floridanus*	109096	2R	Comanja de Corono, Jal, Mexico	1966	68.73	(−)	(+)	(+)	NT
*S*. *floridanus*	in process	15R	Topeka, KS, USA	1988	92.94	(−)	(−)	(−)	(−)
*S*. *floridanus*	146898	16R	Ulysses, KS, USA	1994	38.02	NT	(+)	(+)	(+)
*S*. *floridanus*	163887	3R	Lexington, NE, USA	2005	13.23	(+)	(+)	(+)	(+)
*S*. *floridanus*(*)	163889	4R	Lexington, NE, USA	2005	15.57	(−)	(−)	(−)	(−)
*S*. *floridanus*	163890	6R	Lexington, NE, USA	2005	28.6	(−)	(+)	(+)	(−)
*S*. *floridanus*	163888	11R	Lexington, NE, USA	2005	50.81	(−)	(−)	(−)	(−)
*S*. *audubonii*	147273	18R	Embudo Canyon, NM, USA	2005	265.89	NT	(−)	(−)	(+)

*indicates non-symptomatic negative control. Abbreviations: KS (Kansas), Jal (Jalisco), NE (Nebraska), N.L. (Nuevo Leon), NM (New Mexico), NV (Nevada); NT (not tested). Ratio shows the results of spectrophotometric analysis of DNA templates.

### DNA extraction and amplification

500 **μ**L of lysis buffer (10 mM Tris-HCl, 5 mM EDTA, 200 mM NaCl, 0,2% SDS), was added to a small amount of sample tissue (varying from 11 and 120 mg), which was homogenized, and incubated for 2 h at 60°C with Proteinase K (500 **μ**g/ml). DNA was extracted using a standard phenol-chloroform-isoamyl alcohol technique followed by isopropanol precipitation [35]. The DNA obtained was resuspended in 50 **μ**l H_2_O and stored at −20°C until being tested. All extractions were carried out in a laminar flow cabinet to eliminate contamination risk, and sterile scalpel blades were used for preparation of each sample. Work surfaces were cleaned with 5% bleach between extractions.

DNA was amplified using multiple-primed rolling circle amplification (RCA) [36] with Templiphi 100 Amplification (GE Healthcare) following the manufacturer’s instructions. Multiple primed RCA amplifications were carried out with 0.5 **μ**l of DNA in a total reaction volume of 10 **μ**l. Because of degradation of DNA in archival samples and the lack of information about viral load, the maximum volume recommended by the manufacturer of undiluted DNA was used in all reactions (0.5 **μ**l of template). RCA products were digested with the restriction enzyme *Hind III* (Biotools).

Whole genome amplification of 1–2 **μ**l genomic DNA extractions was performed by Phi29 DNA- polymerase using *Ilustra Ready-to-go genomiPhi V3 DNA amplification* kit (Ge Healthcare). The method is based on multiple isothermal displacement amplification (MDA) [37].

PCR amplification reactions, were done with two pairs of primers designed for this study (Integrated DNA Technologies, Leuven, Belgium): L1CRPVF1 (5´-GGGCAATGGACCACAAAACA-3´) and L1CRPVR1 (5´-TCCTGCCCTGCTGAAGAAATG-3´) for the L1 gene and E7CRPVF1 (5´-TTTCCTTCTGTACTGGCTTTATCG-3´) / E7CRPVR1 (5´-CGCTTACATGGCACGGACACT-3´) for the E7 gene. Each reaction contained 5 μl of total DNA extractions or amplified DNA extractions (MDA), PCR Buffer II 10x (Applied Biosystems), 3 mM MgCl2 (Applied Biosystems), 200 μM dNTPs (Roche), 0.5 μM of each primer, and 0.6 U of DNA polymerase (Ampli-taq, Applied Biosystems) in 25μl of total volume. Amplification reactions were carried out on a MyCycler *Thermal Cycler* (Bio-Rad). Negative controls were included in all reactions and endogenous DNA was evaluated by amplification of newly designed cytochrome b CytrabF1 (5´-CCCCTAYATYGGAACAAC-3´) / CytrabR1 (5´-ATAGGGGTGGAARGGRATTT-3´).

Extraction blanks, PCR negatives and standard negative controls were used in all PCRs and genome amplification, and were consistently negative. There was no evidence of contamination at any stage. This lab was not previously used for work on SfPV. Types within a PV genus typically show less than 60% sequence identity to types of other genera based on global multiple sequence or pairwise alignments of the L1 genes [4]. Thus SfPV strains detected in this study could not result from contamination with PV strains previously used in the lab (*Fringilla coelebs* Papillomavirus, Human Papillomavirus and Bovine Papillomavirus). Positive control used in this study was derived from own samples, and not obtained outside this study.

### Molecular cloning

The vector pUC19, cut with *Hind III* and processed with shrimp alkaline phosphatase (Roche Applied Sciences) to avoid re-ligation, was ligated to the gel purified RCA digested product in a total volume of 10 **μ**l with the T4 DNA ligase (Roche Applied Sciences). One Shot TOPO10 competent *E*. *coli* (Invitrogen) were transformed with the resulting plasmids. The extraction of plasmid DNA from recombinant clones was performed with QIAprep Miniprep Spin kit (Qiagen).

### Sequencing and molecular analysis

Partial sequencing of recombinant clones and PCR products were performed in an ABI Prism 3730 automated sequencer (Perkin Elmer Applied Biosystems, Foster City, CA) at the Genomic Unit of the Scientific Park of Madrid-UCM and in Macrogen Europa. Sequences were confirmed by Sanger sequencing of both strands. Sequences were compared to the GenBank database using the BLAST algorithm.

### Tissue archive and museum samples for pathogen detection

In their excellent review Tsangaras and Greenwood [33] set out seven minimal standard ancient DNA facility criteria, and assess whether 64 studies of pathogens in archival samples met their criteria (such as physically isolated lab work areas, extraction and PCR controls, etc.). Only one study met all seven criteria, and only 26 studies (41%) met four or more criteria. Our study fulfilled 4/7 criteria – better than the majority of previous studies (59%). We did not fulfil criterion 4 (that there should be an inverse correlation between amplification efficiency and length of amplification; the amplicons we obtained were of similar size, so this criterion did not apply in this case); criterion 6 (reproduction of results in a second independent laboratory; this was unnecessary as the weight of evidence suggests contamination did not confound the results obtained); or criterion 7 (cloning of amplification products and sequencing of multiple clones; we sequenced amplicons directly).

### Phylogenetic analysis

The partial E7 sequences (153 bp, excluding primers) obtained were aligned manually with known SfPV1 E7 sequences obtained from GenBank. The best model of nucleotide substitution was selected based on the Akaike (AIC) and Bayesian (BIC) information criteria available in jModeltest 2 software [37]. A PhyML 3.0 [38] maximum likelihood ultrametric tree was built using a heuristic search with the nearest neighbor interchange algorithm for branch swapping, and conducting a bootstrap to estimate statistical support for internal nodes (with 1000 repetitions). As we could only obtain a 131 bp fragment for sample 16R, and the missing end of the fragment involved various polymorphic sites, we did not include it in the analyses used to provide statistical support for the internal nodes of the phylogenetic tree. We later positioned this sample on the tree manually, once we made sure that an analysis based on shorter (131-bp) sequences and including 16R recovered the same topology for the other sequences as the one of the tree built with 153-bp sequences.

### Statistical analysis

Comparisons were made using Fisher’s Exact Test. All *p*-values are two-tailed, and were considered statistically significant if the *p*-value was < 0.05.

## Results

Growths symptomatic for SfPV were detected in 20 individuals from KU (though one was not tested, as explained in methods). All symptomatic individuals were genus *Sylvilagus*, no lesions were detected in either *Brachylagus* or *Lepus* (**Table 2**). 16/580 *S*. *floridanus* (2.8%), 2/281 *S*. *audubonii* (0.7%), and 2/89 *S*. *nuttallii* (2.3%) were symptomatic. *Sylvilagus* (*N* = 20/1096) and *S*. *floridanus* (16/580) were significantly more likely to be symptomatic than *Lepus* (*N* = 0/286; two-tailed, Fisher’s Exact Test, P < 0.05). Comparisons between all other taxa were insignificant with the caveat that numbers of symptomatic individuals are low for analysis. Symptomatic individuals were collected between 1915 and 2005, from five U.S. and two Mexican states (Kansas, Nebraska, Nevada, New Mexico, Texas, Jalisco, and Nuevo Leon; **Table 1**), including 3 symptomatic individuals collected from a small outbreak in south-central Nebraska in 2005. About one third come from samples collected in the previously SfPV1 positive state of Kansas (7 samples); the remainder from locations where SfPV1 has not been identified previously.

**Table 2:** List of Leporidae voucher specimens inspected for SfPV growths in the Kansas University Natural History Museum.

Species	English name	N	S	Pos	% S	% Pos
*Brachylagus idahoensis*	Pygmy Rabbit	13	0	0	0	0
*Lepus alleni*	Antelope Jackrabbit	10	0	0	0	0
*Lepus americanus*	Snowshoe Hare	77	0	0	0	0
*Lepus californicus*	Black-tailed Jackrabbit	80	0	0	0	0
*Lepus callotis*	White-sided Jackrabbit	15	0	0	0	0
*Lepus townsendii*	White-tailed Jackrabbit	91	0	0	0	0
*Sylvilagus aquaticus*	Swamp Rabbit	28	0	0	0	0
*Sylvilagus audubonii*	Desert Cottontail	281	2	1	0.71	0.36
*Sylvilagus bachmani*	Brush Rabbit	21	0	0	0	0
*Sylvilagus brasiliensis*	Forest Cottontail	40	0	0	0	0
*Sylvilagus cunicularius*	Mexican Cottontail	25	0	0	0	0
*Sylvilagus insonus*	Omilteme Cottontail	8	0	0	0	0
*Sylvilagus floridanus*	Eastern Cottontail	580	16^a^	11	2.76	1.89
*Sylvilagus nuttallii*	Mountain Cottontail	89	2	1	2.25	1.12
*Sylvilagus obscurus*	Appalachian Cottontail	1	0	0	0	0
*Sylvilagus palustris*	Marsh Rabbit	35	0	0	0	0
*Sylvilagus transitionalis*	New England Cottontail	1	0	0	0	0
**Total**		1395	20	13	1.43	0.93

Number of individuals inspected (N), symptomatic (S) and PCR positive (Pos) and percentage of individuals’ symptomatic (% S) and of symptomatic individuals positive by PCR (% Pos).

^a^ Note, though 16 *S*. *floridanus* were symptomatic, only 15 were tested. Values of % Pos for *S*. *floridanus* and Leporidae were calculated accordingly.

DNA extracted from cutaneous tumors showed different levels of DNA degradation in electrophoresis gel (data not shown) and spectrophotometric analysis (**Table 1**). Ten less degraded DNA extractions were amplified by RCA, and digested by Hind III, although specific band sizes compatible with SfPV1 digested with this restriction enzyme (around 4kb, 3kb, and 500b) were only seen (**S1 Fig**) in two (3R and 7R). The 4Kb band from only sample 3R could be cloned in the pUC19 vector. The resulting sequence of 1000 bp from both extremes of the cloned sequenced confirmed it was SfPV1. The two fragments of 1080 bp and 990 bp showed > 99% identity to L2 gene (GenBank Acc. No. KP202721; 1478 bp) and to E1 gene (GenBank Acc. No. KP202722; 1808 bp) respectively, from CRPVa4 (accession No AJ404003) isolated in *S*. *floridanus* in Kansas [21]. Subsequently all samples were amplified by PCR using specific primers for the L1 and E7 regions of SfPV1 that amplified small fragments of 179 and 208 bp respectively (including primers), particularly suitable for degraded samples. Both amplifications were coincident and PCR positive in 7 from 19 samples (36.8%; **S2** and **S3 Figs**). No amplification was obtained from negative control 4R, the sample from the asymptomatic *S*. *floridanus*.

All samples that tested negative by PCR were subjected to whole genome amplification using MDA followed by L1-PCR. Positive amplifications were obtained from 5 samples previously negative by direct PCR (samples 5R, 8R, 10R, 18R, and 20R; **S4 Fig**). Again, no amplification was obtained from the asymptomatic rabbit (4R). In addition some previously positive PCR samples were included (3R, 6R, 13R, and 16R) but only three (3R, 13R, and 16R) could be amplified with L1 primers corroborating previous results. 12/19 samples (63.2%) tested SfPV1 positive at least once using PCR-L1, either from original DNA extracts or PCR-L1 of the Phi29 polymerase amplification product. A second DNA extraction was carried out on all negative samples for which sufficient material remained (9R, 11R, 15R, 17R, 19R) and one previously positive sample (5R). These samples were then challenged with L1/E7 PCR. All with exception of 5R were negative again (data not shown). Negative control sample (4R) was positive when challenged with cytochrome b primers. All other SfPV1 negative samples were negative.

We note that positive amplification was obtained in seven of the 12 oldest samples (from 1915 to 1964), including the oldest sample analyzed (7R) from 1915—a full century ago. Direct PCR only amplified 23% of samples (3/12) from this period, but the amplification with Phi29 polymerase followed by L1-PCR improved the positive results (58%). By comparison, direct amplification was obtained from five of seven (71%) more recent samples (1966–2005) and the two negative samples were not amplified by Phi29 polymerase followed by L1-PCR. Specimen (7R) collected in 1915 from southeastern, Kansas is the oldest confirmed case of SfPV1 to date.

A total of 19 cottontail rabbits were tested, corresponding to three species of *Sylvilagus*: *S*. *floridanus* (15 samples), *S*. *audubonii* (2 samples), and *S*. *nuttallii* (mountain cottontail, 2 samples). SfPV1 infection has been confirmed in at least one individual from the three symptomatic species in the museum collection. However, samples from *S*. *audubonii* and *S*. *nuttallii* tested positive for PV only following MDA amplification, presumably due to DNA degradation or low viral yield. There was no significant difference in the number of positive individuals of each species, though numbers of positive individuals are low for analysis.

**Fig 3:**
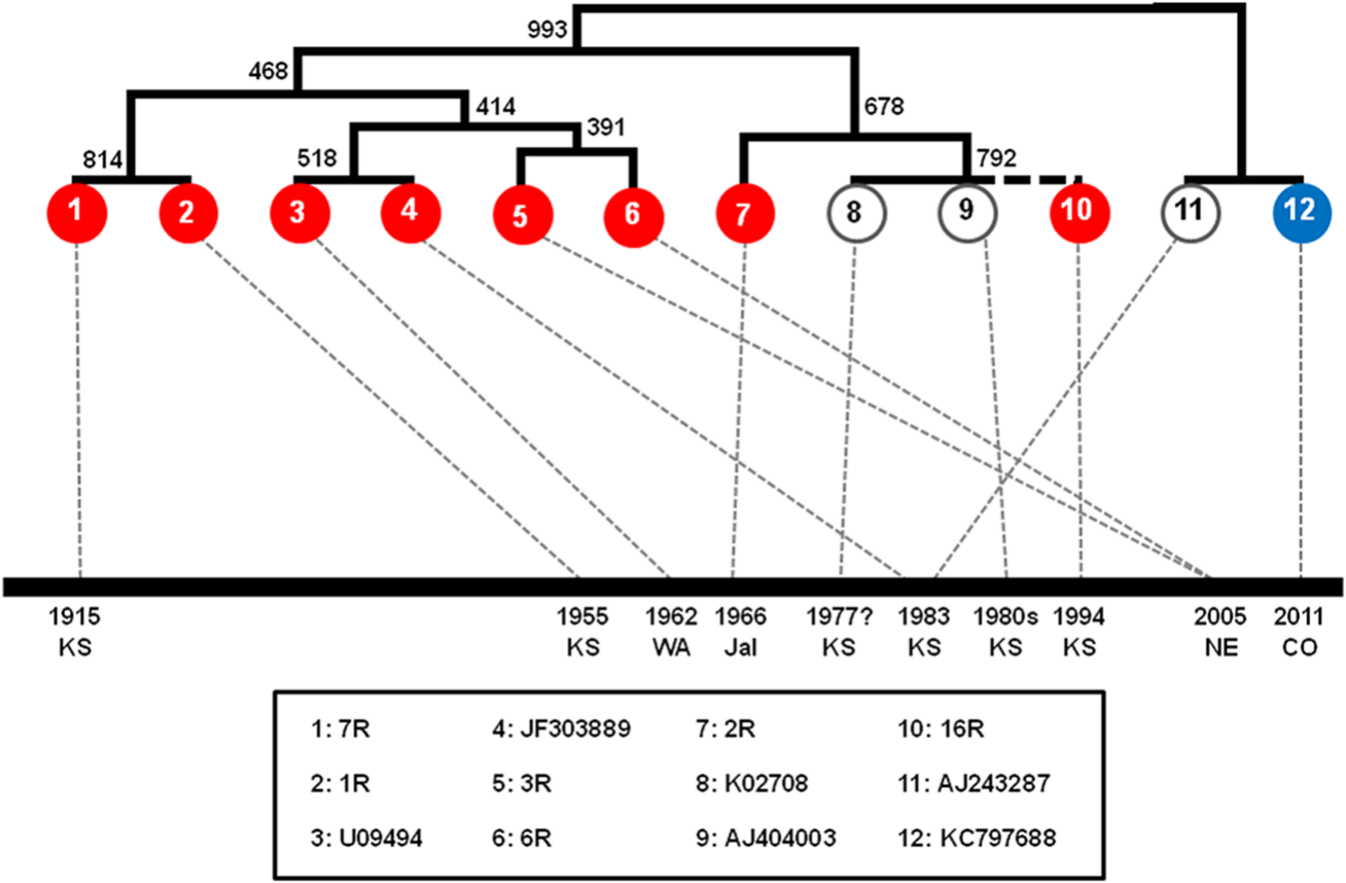
Maximum likelihood tree of partial E7 SfPV 1 sequences. The tree was constructed using six 153 bp E7 sequences obtained in our study (1R, 2R, etc.), supplemented with six sequences from previous studies (GenBank accession numbers shown), along with locality and date. Host species is indicated by color (blue = S. *audubonii*, red = *S*. *floridanus*, white = *Sylvilagus* sp.). Numbers indicate bootstrap support for internal nodes (with 1000 repetitions). Partial sequence 16R was manually added to its closest relatives (resolved sequence was 100% identical to K02708 and AJ404003), but it was excluded from the bootstrap analysis. The assumed position of sequence 16R is represented with a dashed line.

The viral L1 sequence (179 bp) from isolates with the stronger signal (1R, 2R, 3R, and 16R) corresponding to *S*. *floridanus* showed 100% identity to previous published SfPV1a sequences [11,19,20], all from Kansas. In contrast, E7 sequence (153 bp, excluding primer sequences) from isolates 1R, 2R, 3R, 6R, 7R, and 16R showed some differences (**Fig 3** and **S5 Fig**). Partial E7 sequences generated in this study, below the minimum sequence length for accession to GenBank, are shown in **S1** and **S2 Tables**. Both AIC and BIC selected Jukes–Cantor (JC69) as the best model of nucleotide substitution for the alignment of E7 sequences, with a significant proportion of invariable sites. The maximum likelihood tree (built with the JC+i model of nucleotide substitution) showed low support for most internal nodes, as was expected due to the short length of the alignment, but still allowed the provisional placement of sequences obtained in this study into a broader phylogenetic framework. Sequences 1R and 7R from northcentral (1955) and southeastern (1915) Kansas share 100% identity. The 3R and 6R sequences (both southcentral Nebraska, 2005) were most closely related to one another and grouped with JF303889 (Hershey strain) [20] from Kansas, 1980s (county not provided; identity > 99%), and the identical U09494 (Whidbey Island, Washington state, 1962; USNM 567614). The resolved region of 16R (southwestern Kansas 1994; just 131 bp), was identical to AJ404003 (a4 strain), from southcentral Kansas (1983 [21]), and CTPV “Shope” K02708 [19], also southcentral Kansas (collected at some point between 1966–1977, pers. comm. G. Orth, 30 October 2014). 2R (Jalisco, Mexico, 1966) showed similarity (98.7%) to this group. The subtype b sequence SfPV1 type B AJ243287 [11], also southcentral Kansas (1983) and KC797688, from southeastern Colorado (from *S*. *audubonii*, 2010 [12]) are identical. It is noticeable that each sister group occurs within a 45 year period, although the topology of the tree implicates the presence of representatives of each major virus lineage throughout the study period. One clade has not been observed since 1955, a second has not been observed since 1994, while the other two have been observed in the last decade. The three recent clades overlap temporally. All clades occur sympatrically, in Kansas at least. Three groups are relatively close geographically, locality of detection is ca < 600 km (Cherokee and Smith counties, Kansas; Dawson County, Nebraska, and Kansas (county unknown); Kingman County, Kansas and Larimer County, Colorado). Only the identical (for this partial E7 sequence) K02708 and AJ404003, both from southcentral Kansas, and 3R and 6R from southeastern Colorado, were geographically closest to the sequence with which they shared highest identity. The remaining group contains cases from Grant and Kingman counties, Kansas and Jalisco, Mexico, which are approximately 1900 km apart.

## Discussion

We directly amplify 7 of 19 (36.8%) SfPV symptomatic museum rabbit specimens using PCR. Using additional amplification techniques (RCA and MDA plus PCR), we obtained evidence of infection in 63.2% of samples, including some samples that produced a faint signal unsuitable for sequencing and one sample 100 years old. Most of these second positive samples correspond to samples collected pre-1950, probably containing fragmented DNA. MDA amplification improves detection rates in low-copy and highly degraded DNA for forensic testing [38], though it has not been extensively used in archived samples. Our improved success rate may be due to the use of Phi29 polymerase in both MDA and RCA amplification [39]; if viral DNA is fragmented it is amplified by MDA, and if it is circular and complete, but present in low quantities, it is amplified by RCA. Most samples were amplified by PCR both directly and following Phi29-amplification, with the exception of sample 6R, which was amplified directly, but not following Phi29-amplification. The amplification product for 6R produced a strong band in the gel (**S2, S3 and S4 Figs**), suggesting that excessive amplification of DNA may have inactivated the polymerase. Unlike PCR amplification, the proofreading 3´–5´ exonuclease activity of Phi29 polymerase ensures high fidelity amplification. Possibly the use of more advanced technology including high-throughput shotgun sequencing [33,40], combined to MDA or RCA amplification, could provide improvements to the number of sequences obtained or the percentage of positive samples.

Negative results after direct PCR or MDA/PCR in symptomatic specimens do not necessarily constitute evidence of absence of papillomavirus infection. The preservation of host DNA and the lack of inhibition of polymerase should be confirmed prior to testing, although in many studies it has not [33]. If it is possible to confirm there is no inhibition, a differential diagnosis could be undertaken in negative samples, including Rabbit Oral Papillomavirus, which would not be amplified by the primers used in this study, Shope fibromavirus or Myxoma virus, both caused by two leporipoxviruses (*Poxviridae*), for which the clinical presentation is slightly different [10]. It is probable that all symptomatic animals tested in this study were SfPV1 positive. All samples negative in this study also failed to amplify *Sylvilagus* cytochrome b. Inability to amplify DNA from older specimens is likely explained by age of the sample. However, it was surprising that two extractions of samples 11R and 15R were negative for SfPV1, as each individual had numerous lesions, visually identical to those of positive individuals, and both samples were collected recently (2005 and 1988, respectively).

**Fig 4:**
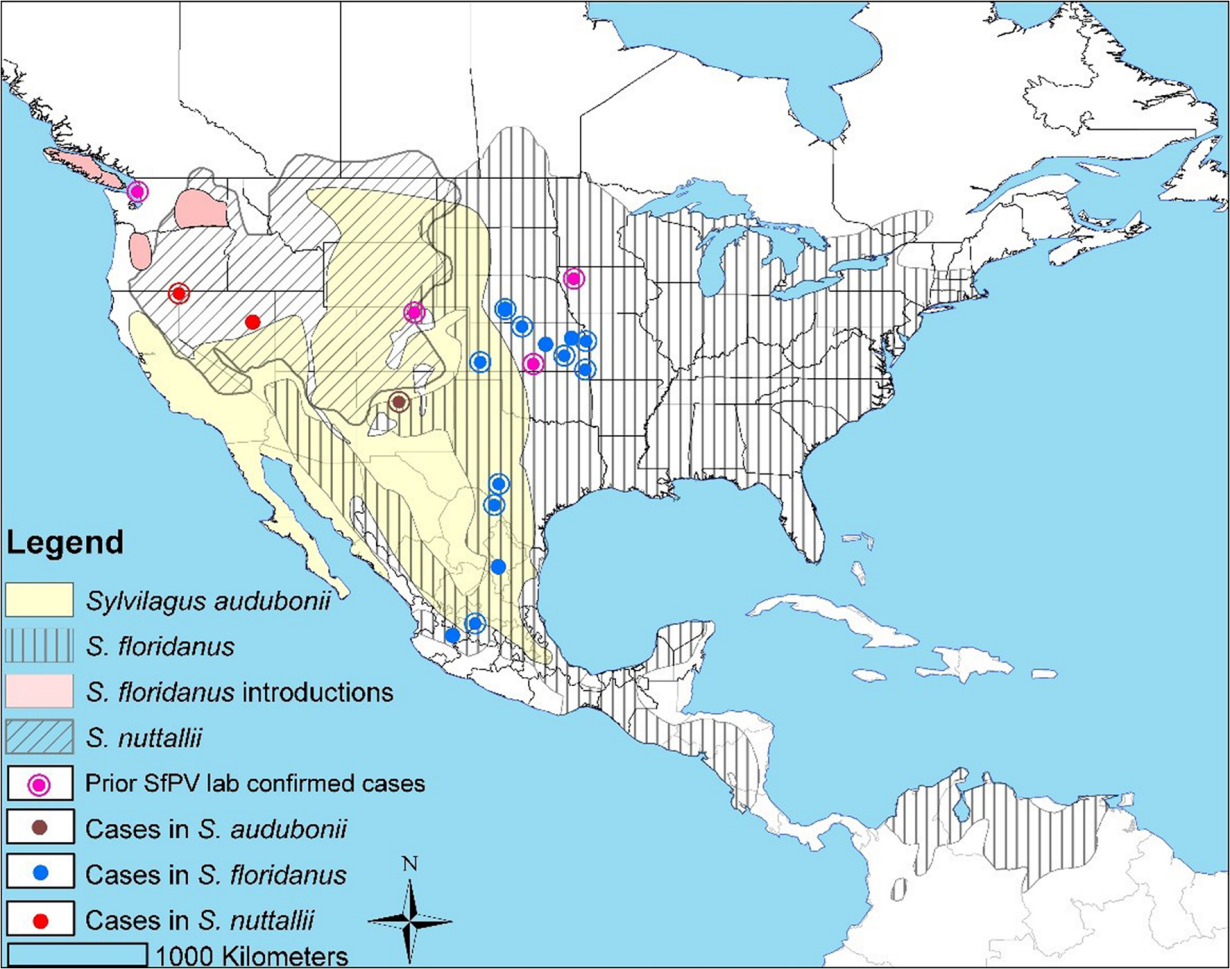
Distribution of the SfPV1 cases and three known *Sylvilagus* hosts. Host distributions are depicted with different shading [16]. Similarly SfPV case locations are color coded for host species, etc. See legend for details. Outer circles to points indicate SfPV positive samples, their absence indicates symptomatic individuals. We omit the locality of one symptomatic *S*. *audubonii* and CRPV Hershey “Kansas” (GenBank Acc. No: JF303889), for which locality information was missing or vague.

The natural hosts for SfPV infection are cottontail rabbits of genus *Sylvilagus* although precise knowledge of the taxonomic range of SfPV host species is not well known. We confirm the presence of SfPV1 in three partially sympatric species of *Sylvilagus*, two of which (*S*. *floridanus* and *S*. *audubonii*) had previously been described as SfPV1 hosts (**Fig 4**). SfPV1 infection in the mountain cottontail rabbit (*S*. *nuttallii*) has not been recognized previously. All three species of *Sylvilagus* are closely related [41]. Previous laboratory infection studies [9] have shown that SfPV can infect other leporids, so it is unsurprising that an additional *Sylvilagus* species should be susceptible in the wild. Our results support the hypothesis that SfPV1 is not strictly associated with *Sylvilagus floridanus*, and provide evidence for natural infection of two additional *Sylvilagus* species. Prevalence rates did not differ significantly in the three positive species. However, we advise caution in interpreting these data, as representation of most negative specimens was low for meaningful comparison, and sampling, at least of *S*. *floridanus*, was biased. All recent infected *S*. *floridanus* (N = 4) were deliberately collected because they were symptomatic, and we assume that interesting “warty rabbits” were more likely to be retained for the KU collection than an ordinary rabbit. On the other hand, many specimens with inconspicuous growths would not have been retained for the collection. However, while general prevalence data may be biased, there is no reason to have sampled lesion-positive individuals more frequently in one species than in others. This suggests that prevalence rates of symptomatic individuals in *Sylvilagus* and *S*. *floridanus* are indeed significantly greater than in *Lepus*. All specimens were inspected visually and palpated. It is possible, though examination of specimens was very thorough, that small lesions were missed when obscured by fur. A single (missed) finding among species with less than 50 individuals might change the conclusions, though obviously the probability of false negatives is, *a priori*, no lower in species with a large sample size than a smaller one. A systematic screening of asymptomatic specimens, or better from freshly sampled wild individuals, might obtain a better estimate of SfPV prevalence, though it is beyond the scope of this study.

Our results extend the known natural distribution of SfPV1 to several U.S. states outside the 1970 estimated range (**Fig 1**; Nevada, New Mexico, and Texas), as well as the first positive SfPV1 sequence from Mexico, from one *S*. *floridanus* captured in Jalisco. Our findings provide the first evidence for SfPV1 presence to the west of the Rocky Mountains (from 1949)—long before the detection of SfPV1 in *S*. *floridanus* translocated to Washington State [13]—and west of the Sierra Madre Occidental (1966). This increases the “potential range” of SfPV, by which we mean the area inhabited by competent SfPV hosts, as we add *S*. *nuttallii* to the list of hosts, and hence the *S*. *nuttallii* range, to the ranges of *S*. *audubonii* and *S*. *floridanus*. We detected this rare virus broadly in this “potential range”, to the west of the Missouri River, where the KU collections are strongest. We are unable to confirm in this study that SfPV is found to the east of the Missouri River, though a symptomatic *S*. *floridanus* from Maryland is housed at the Smithsonian Institution (U.S. National Museum of Natural History; R.M. Timm, unpubl. data), supporting the hypothesis that SfPV was transmitted broadly within the native range of SfPV host species. Further study is required to accurately describe SfPV presence.

Defining the current and historic range of SfPV transmission is complicated by the transportation of *Sylvilagus* from SfPV hotspots (especially Kansas) to many areas of the continental U.S. of unknown SfPV status, which was extensive from the 1920s to 1950s, and persisted at least into the 1970s [15]. The current distribution is anticipated to be broader than the pre-transportation distribution hypothesized in **Fig 1**. The Whidbey Island, WA case demonstrates that SfPV1 has been transported outside its range. Our results, do not confirm new cases in the East and Northwest, though the KU collection contains few individuals from those areas. However, we detect several historic cases from the Southwest and Mexico—outside the known distribution—regions not known to have received transported rabbits. This suggests that the distribution hypothesized in **Fig 1** underestimated the true historic distribution, possibly because only *S*. *floridanus* was considered to host SfPV. Two recent cases from Larimer County, Colorado [10,12]—also outside the 1980 hypothesized range—show that either Shope underestimated the distribution, or that it has expanded since 1970.

SfPV clearly circulates in three *Sylvilagus* species, albeit closely related species. Both SfPV1 strains are highly similar (97% genomic identity), and thus do not constitute distinct viral species. However, more data are necessary to establish whether all SfPV1 lineages circulate freely in each *Sylvilagus* species or are specific to a single host species. Current sequence data can only confirm SfPV1a from *S*. *floridanus* and SfPV1b from *S*. *audubonii*, though data are incomplete and we cannot discount broader host range for either. We did not obtain good quality sequence data from *S*. *nuttallii*. Informative genetic sequences were small and obtained from only six individuals of *S*. *floridanus*. This information did not provide sufficient information for detailed analysis of strain variation in space or time. Phylogenetic analyses of twelve sequences (six from this study and six from previous studies), confirm that two strains are identical to sequences collected 28 and 40 years previously–suggesting at least some strains are conservative. Some strains appear to circulate sympatrically, at least within Kansas.

The E7 sequence we show for SfPV1 is quite conservative. For instance, twelve samples from different host individuals yield only seven unique sequences, including from two sequences (1R and 7R) which were collected forty years apart. On the other hand, they are not immutable: two sequences (3R and 6R) recovered from southcentral Nebraska in 2005, are distinct, albeit one another’s sister taxon. Although SfPV1a sequences are closely related, the relationship between sequences is not linear in time–that is to say sequence 1R, collected in 1915 is not ancestor to sequence 10R collected in 1994; neither are ancestor to sequence 3R collected in 2005. Rather all share a common ancestor that must have existed prior to 1915. Thus, the diversity of SfPV1 viruses appears to be consistently fairly high, and those strains are generally conservative (over several decades), rather than describing a situation in which one strain rapidly replaces another.

In conclusion, historical museum specimens can provide extremely valuable sources for detecting diseases in wildlife as well as delineating hosts and geographic and temporal range–in this case including a sequence that is a century old. They provide an historic perspective that the present-time sampling cannot provide. Broad survey of natural history collections may be necessary because of the difficulty in working with archived DNA, in terms of sequence length, the effort required to obtain data, and the suspicion that some samples test false negative. Future work on historic SfPV from museums will probably yield lineage detection in most samples, particularly if multiple methodologies, especially RCA and MDA-PCR, are used in tandem. It would be desirable to obtain good quality sequence data–preferably whole genomes–from any *S*. *audubonii* and *S*. *nuttallii*, and from *S*. *floridanus* originating outside Kansas. This would improve efforts to characterize SfPV diversity, map SfPV lineage to host species, locality, and time, and demonstrate the existence or absence of SfPV-host co-evolution. Ideally new SfPV samples should be collected from living rabbits or fresh frozen specimens. Future studies should ensure that high quality data is provided for hosts as a minimum, host species, date, and exact locality of capture, and museum voucher number and sequences posted on an appropriate database (such as GenBank). Samples from different individuals, localities, species, should not be pooled because valuable descriptions for the study of viral evolution or ecology will be lost as we have observed in the historical reports of Shope’s papillomavirus.

## Supporting Information

S1 Fig SfPV RCA digested with EcoRI. **(M) λ Hind III marker.** Lines 1 and 2: samples 3R and 7R respectively. White arrows: digested products from rolling amplification. Blue arrows: RCA concatemerized product with higher molecular weight.
(TIF)

S2 Fig SfPV L1-PCR. (M) Ladder- plus. **Row A**: samples 1R to 11R. **Row B**: Lanes 1–9 samples 12R to 20R, lane 10 positive PCR control, lane 11 negative PCR control.
(TIF)

S3 Fig SfPV E7-PCR. (M) Ladder-plus. **Row A**: samples 1R to 13R; **Row B**: Lanes 1–7 samples 14R to 20R, lane 8 positive PCR control, lane 9 negative PCR control.
(TIF)

S4 Fig SfPV (MDA + L1-PCR). **Row A**: lanes 1–10 (samples 4R, 5R, 8R, 10R, 11R, 12R, 3R, 6R, 9R); lane 11 negative PCR control. **Row B**: lanes 1–8 (samples 14R, 15R, 17R, 18R, 19R, 20R, 13R, 16R), lane 9 positive PCR control, lane 10 (negative PCR control).
(TIF)

S5 Fig Minimum spanning haplotype network of partial E7 SfPV 1 sequences. Each line or dot on lines represents a mutational step, and the size of circles stands for number of sequences sharing haplotype as indicated in the inset. The data are the same as those used to build the tree shown in Fig 3: six 153 bp E7 sequences obtained in our study (1R, 2R, etc.), plus six sequences from previous studies (GenBank accession numbers shown). Host species is indicated by colour (blue = S. audubonii, red = S. floridanus, white = Sylvilagus sp.). Sequence 16R is identical to K02708 and AJ404003 for the 131 bp available for all sequences, and is included in parenthesis to show that identity is assumed.
(TIF)

S1 Table Nucleotide-level comparison of partial E7 sequences for SfPV1, includes 6 sequences generated herein. (DOCX)

S2 Table Partial E7 sequences generated in this study; partial E7 sequences from this study were not accessioned to GenBank as they are <200 bp—the minimum sequence length required by GenBank. (DOCX)
