# Supplementary figures and images for: A Century of Shope Papillomavirus in Museum Rabbit Specimens
Source: PLoS One. 2015 Jul 6;10(7):e0132172. doi: 10.1371/journal.pone.0132172 (PMC4493010; doi:10.1371/journal.pone.0132172)

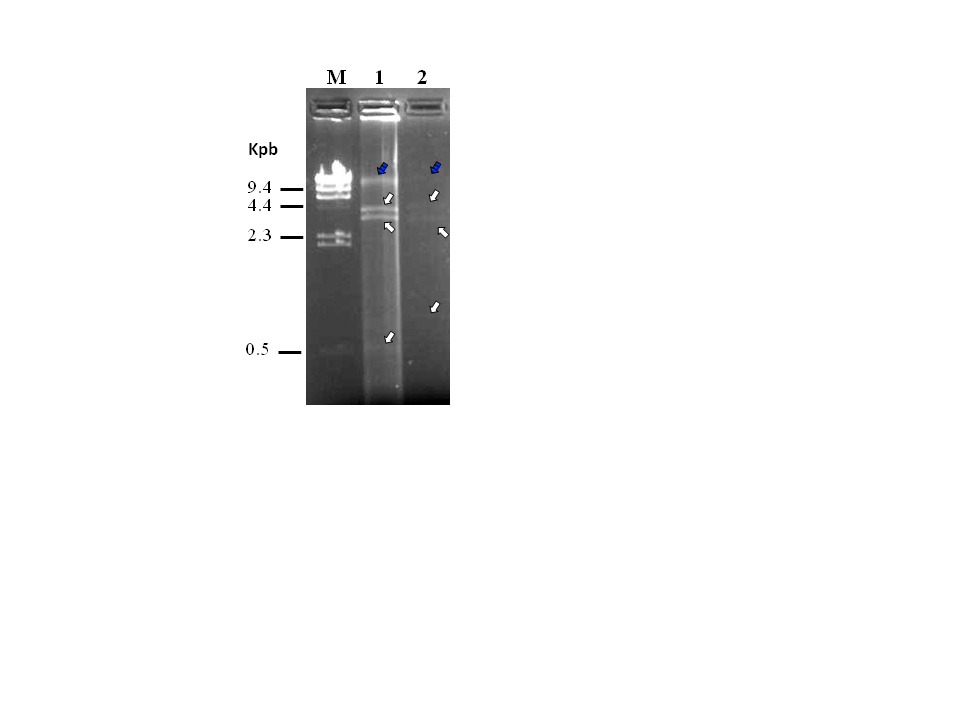

Supplement: S1 Fig — (M) λ Hind III marker. Lines 1 and 2: samples 3R and 7R respectively. White arrows: digested products from rolling amplification. Blue arrows: RCA concatemerized product with higher molecular weight. (TIF) [file pone.0132172.s001.tif]

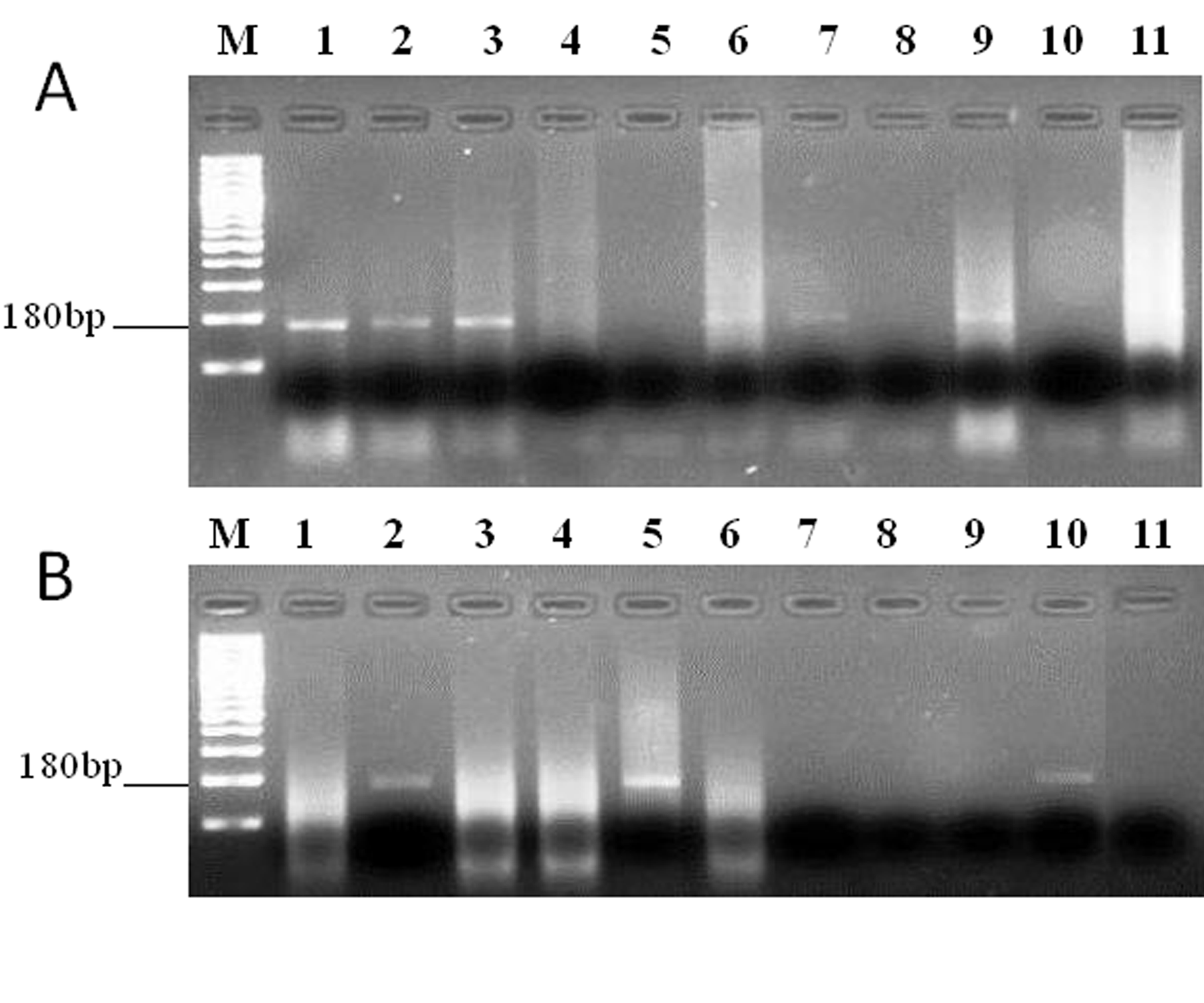

Supplement: S2 Fig — (M) Ladder- plus. Row A: samples 1R to 11R. Row B: Lanes 1–9 samples 12R to 20R, lane 10 positive PCR control, lane 11 negative PCR control. (TIF) [file pone.0132172.s002.tif]

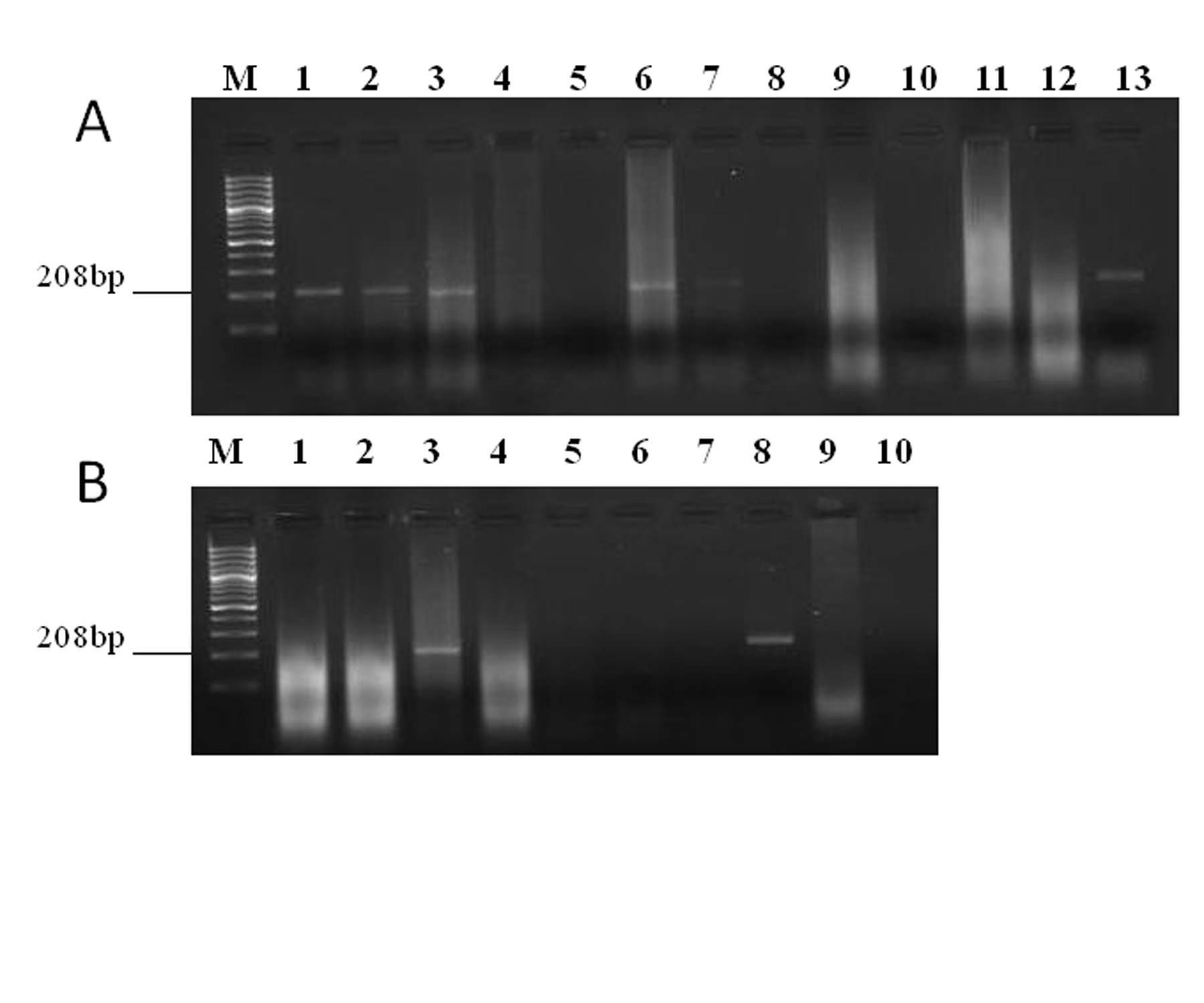

Supplement: S3 Fig — (M) Ladder-plus. Row A: samples 1R to 13R; Row B: Lanes 1–7 samples 14R to 20R, lane 8 positive PCR control, lane 9 negative PCR control. (TIF) [file pone.0132172.s003.tif]

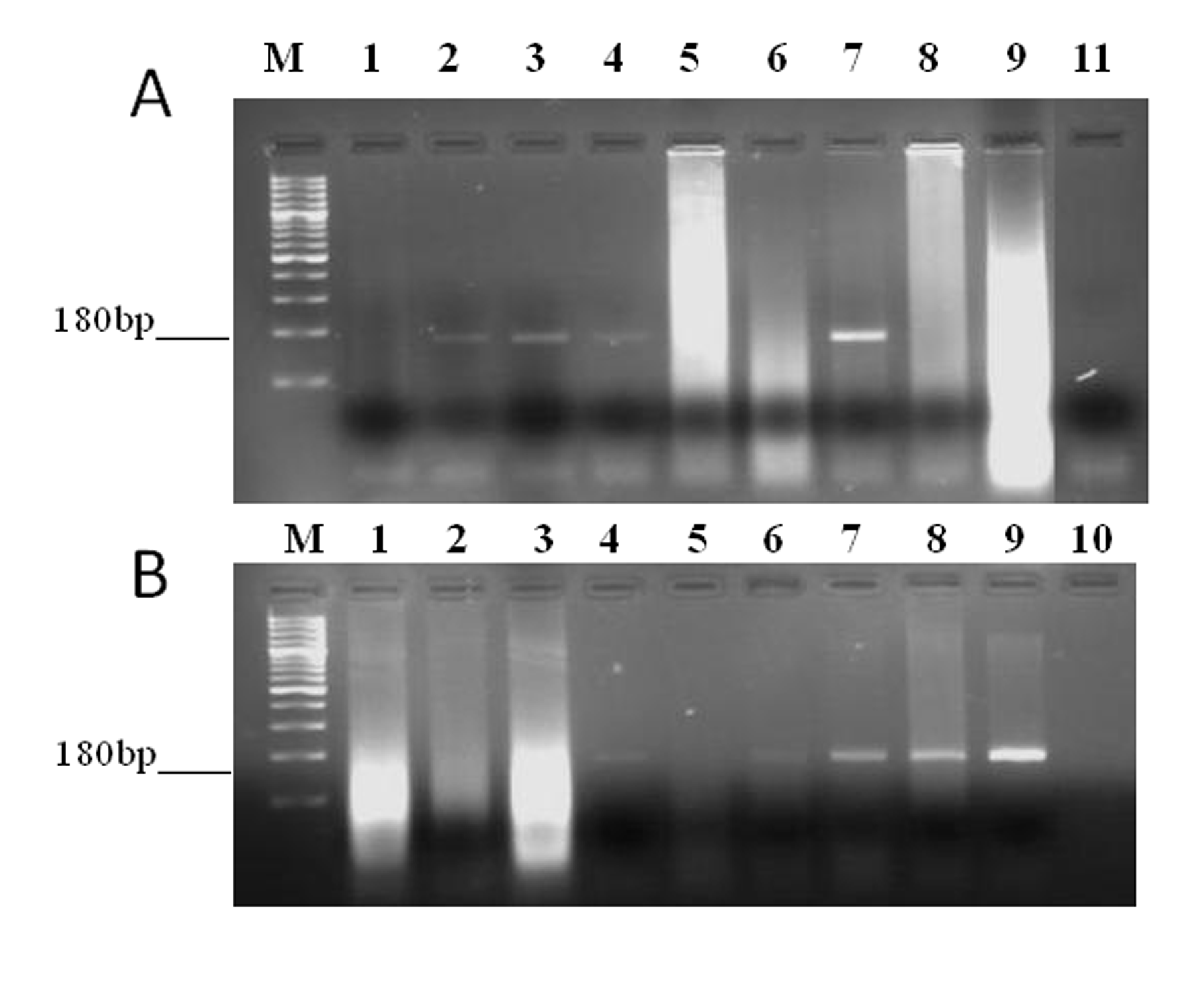

Supplement: S4 Fig — Row A: lanes 1–10 (samples 4R, 5R, 8R, 10R, 11R, 12R, 3R, 6R, 9R); lane 11 negative PCR control. Row B: lanes 1–8 (samples 14R, 15R, 17R, 18R, 19R, 20R, 13R, 16R), lane 9 positive PCR control, lane 10 (negative PCR control). (TIF) [file pone.0132172.s004.tif]

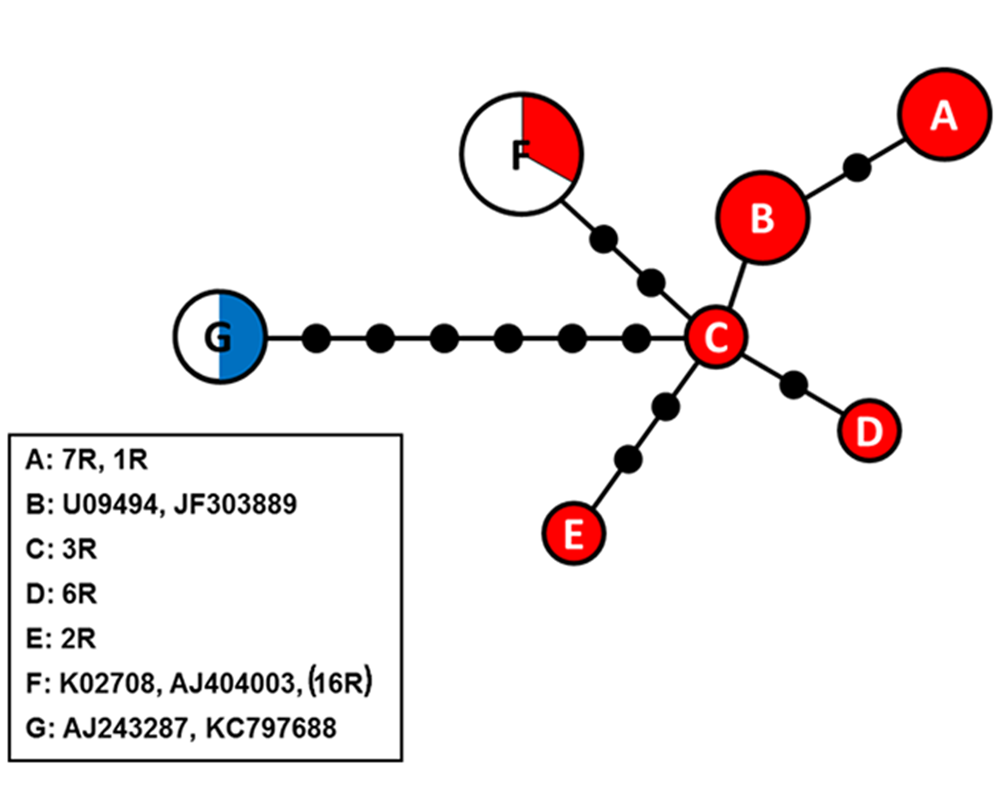

Supplement: S5 Fig — Each line or dot on lines represents a mutational step, and the size of circles stands for number of sequences sharing haplotype as indicated in the inset. The data are the same as those used to build the tree shown in Fig 3: six 153 bp E7 sequences obtained in our study (1R, 2R, etc.), plus six sequences from previous studies (GenBank accession numbers shown). Host species is indicated by colour (blue = S. audubonii, red = S. floridanus, white = Sylvilagus sp.). Sequence 16R is identical to K02708 and AJ404003 for the 131 bp available for all sequences, and is included in parenthesis to show that identity is assumed. (TIF) [file pone.0132172.s005.tif]
