# Supplementary material for: A Century of Shope Papillomavirus in Museum Rabbit Specimens
Source: PLoS One. 2015 Jul 6;10(7):e0132172. doi: 10.1371/journal.pone.0132172 (PMC4493010; doi:10.1371/journal.pone.0132172)
Supplement: S1 Table — (DOCX) [file pone.0132172.s006.docx]

**Table S1. Nucleotide-level comparison of partial E7 sequences for SfPV1,** includes 6 sequences generated herein.

| **SAMPLE** | **0’ 50’** |
| --- | --- |
| 1R | ATGATAGGCA GAACTCCTAA GCTTAGTGAG CTGGTTTTAG GTGAAACTGC |
| 2R | ---------- ---------- ---------- ---------- ---------- |
| 3R | ---------- ---------- ---------- ------C--- ---------- |
| 6R | ---------- ---------- ---------- ------C--- ---------- |
| 7R | ---------- ---------- ---------- ---------- ---------- |
| 16R | ---------- ---------- ---------- ---------- ---------- |
|  | **51’ 100’** |
| 1R | TGAAGCGCTT AGTCTGCATT GCGACGAATC ATTAGAGAAT TTAAGTGATG |
| 2R | ---------- ---------- --------G- ---------- ---------- |
| 3R | ---------- ---------- --------G- ---------- ---------- |
| 6R | ---------- ---------- --------G- ---------- ---------- |
| 7R | ---------- ---------- ---------- ---------- ---------- |
| 16R | ---------- ---------- --------G- ---------- ---------- |
|  | **101’ 150’** |
| 1R | ATGATGAGGA GGATCATCAA GATAGACAGG TGCACAGAGA AAGGCCCTAT |
| 2R | ---------- ---------- -------C-- --T---T--- ---------- |
| 3R | ---------- ---------- ---------- ------T--- ---------- |
| 6R | ---------- ---------- -------G-- ------T--- T--------- |
| 7R | ---------- ---------- ---------- ---------- ---------- |
| 16R | ---------- ---------- ----------- |
|  | **151’ 174’** |
| 1R | GCAGTGTCCG TGCCATGTAA GCG |
| 2R | ---------- --- |
| 3R | ---------- ---------- --- |
| 6R | ---------- ------- |
| 7R | ---------- ---------- ---- |
| 16R |  |
