# Supplementary material for: A Century of Shope Papillomavirus in Museum Rabbit Specimens
Source: PLoS One. 2015 Jul 6;10(7):e0132172. doi: 10.1371/journal.pone.0132172 (PMC4493010; doi:10.1371/journal.pone.0132172)
Supplement: S2 Table — (DOCX) [file pone.0132172.s007.docx]

**Table S1. Partial E7 sequences generated in this study**; partial E7 sequences from this study were not accessioned to GenBank as they are <200 bp—the minimum sequence length required by GenBank.

| **SAMPLE** | **SIZE (bp)** | **SEQUENCE (5´-3´)** |
| --- | --- | --- |
| 1R | 173 | ATGATAGGCAGAACTCCTAAGCTTAGTGAGCTGGTTTTAGGTGAAACTGCTGAAGCGCTTAGTCTGCATTGCGACGAATCATTAGAGAATTTAAGTGATGATGATGAGGAGGATCATCAAGATAGACAGGTGCACAGAGAAAGGCCCTATGCAGTGTCCGTGCCATGTAAGCG |
| 2R | 163 | ATGATAGGCAGAACTCCTAAGCTTAGTGAGCTGGTTTTAGGTGAAACTGCTGAAGCGCTTAGTCTGCATTGCGACGAAGCATTAGAGAATTTAAGTGATGATGATGAGGAGGATCATCAAGATAGACCGGTGTACATAGAAAGGCCCTATGCAGTGTCCGTGC |
| 3R | 173 | ATGATAGGCAGAACTCCTAAGCTTAGTGAGCTGGTTCTAGGTGAAACTGCTGAAGCGCTTAGTCTGCATTGCGACGAAGCATTAGAGAATTTAAGTGATGATGATGAGGAGGATCATCAAGATAGACAGGTGCACATAGAAAGGCCCTATGCAGTGTCCGTGCCATGTAAGCG |
| 6R | 167 | ATGATAGGCAGAACTCCTAAGCTTAGTGAGCTGGTTCTAGGTGAAACTGCTGAAGCGCTTAGTCTGCATTGCGACGAAGCATTAGAGAATTTAAGTGATGATGATGAGGAGGATCATCAAGATAGACGGGTGCACATAGATAGGCCCTATGCAGTGTCCGTGCCATG |
| 7R | 174 | ATGATAGGCAGAACTCCTAAGCTTAGTGAGCTGGTTTTAGGTGAAACTGCTGAAGCGCTTAGTCTGCATTGCGACGAATCATTAGAGAATTTAAGTGATGATGATGAGGAGGATCATCAAGATAGACAGGTGCACAGAGAAAGGCCCTATGCAGTGTCCGTGCCATGTAAGCGA |
| 16R | 131 | ATGATAGGCAGAACTCCTAAGCTTAGTGAGCTGGTTTTAGGTGAAACTGCTGAAGCGCTTAGTCTGCATTGCGACGAAGCATTAGAGAATTTAAGTGATGATGATGAGGAGGATCATCAAGATAGACAGGT |
